# Compression and contact area of anterior strut grafts in spinal instrumentation: a biomechanical study

**DOI:** 10.1186/1471-2474-14-254

**Published:** 2013-08-26

**Authors:** Antonius Pizanis, Jörg H Holstein, Felix Vossen, Markus Burkhardt, Tim Pohlemann

**Affiliations:** 1 Department for Trauma-, Hand- and Reconstructive Surgery, University Medical Centre of the Saarland, Homburg, Saar, D 66421, Germany

**Keywords:** Graft compression, Anterior fixation, Posterior fixation, Spine biomechanics

## Abstract

**Background:**

Anterior bone grafts are used as struts to reconstruct the anterior column of the spine in kyphosis or following injury. An incomplete fusion can lead to later correction losses and compromise further healing. Despite the different stabilizing techniques that have evolved, from posterior or anterior fixating implants to combined anterior/posterior instrumentation, graft pseudarthrosis rates remain an important concern. Furthermore, the need for additional anterior implant fixation is still controversial. In this bench-top study, we focused on the graft-bone interface under various conditions, using two simulated spinal injury models and common surgical fixation techniques to investigate the effect of implant-mediated compression and contact on the anterior graft.

**Methods:**

Calf spines were stabilised with posterior internal fixators. The wooden blocks as substitutes for strut grafts were impacted using a “pressfit” technique and pressure-sensitive films placed at the interface between the vertebral bone and the graft to record the compression force and the contact area with various stabilization techniques. Compression was achieved either with posterior internal fixator alone or with an additional anterior implant. The importance of concomitant ligament damage was also considered using two simulated injury models: pure compression Magerl/AO fracture type A or rotation/translation fracture type C models.

**Results:**

In type A injury models, 1 mm-oversized grafts for impaction grafting provided good compression and fair contact areas that were both markedly increased by the use of additional compressing anterior rods or by shortening the posterior fixator construct. Anterior instrumentation by itself had similar effects. For type C injuries, dramatic differences were observed between the techniques, as there was a net decrease in compression and an inadequate contact on the graft occurred in this model. Under these circumstances, both compression and the contact area on graft could only be maintained at high levels with the use of additional anterior rods.

**Conclusions:**

Under experimental conditions, we observed that ligamentous injury following type C fracture has a negative influence on the compression and contact area of anterior interbody bone grafts when only an internal fixator is used for stabilization. Because of the loss of tension banding effects in type C injuries, an additional anterior compressing implant can be beneficial to restore both compression to and contact on the strut graft.

## Background

Surgical spinal fracture repair can be achieved via numerous techniques employing a posterior, anterior or combined approach. In unstable spinal fracture cases, in which the weight bearing role of the anterior column is compromised, anterior reconstruction is required, using either iliac crest autograft or a distractable vertebral body implant in addition to stabilizing implants. Most surgeons use distractible implants combined with a cancellous bone or allograft to bridge bi-segmental lesions, whereas fractures limited to one injured segment and disc space can be treated by monosegmental fusion through bone strut grafts [1]. The relatively high rate of pseudarthrosis following anterior strut grafting of 17-35% [2-4] has caused much debate amongst surgeons as to if and when additional anterior implants should be used to secure the bone grafts. Since bony non-union can have either a biological or biomechanical etiology, it is important to elucidate the role of implants on anterior bone grafts in driving the biomechanical causes of pseudarthrosis in these spinal trauma patients.

Bony fusion is essential in order to preserve the initial reduction obtained from the surgical procedure. Numerous factors can have an effect on bone fusion rates in spinal trauma repair. While many studies have examined the biomechanics of a range of instrumentation for spinal fixation, these studies limit their focus to corpectomy surgical models [5-8]. Some surgeons prefer the additional use of anterior locking screw-plates or screw-rods in conjunction with the common posterior fixation implants. However, while these additional implant devices might support the fixation and maintain reduction in the spine, they can also increase the stiffness at the segmental level. Consequently, concerns have been expressed that rigid fixation, such as that seen with static anterior plating, may result in graft stress shielding, thereby reducing the mechanical load that is necessary for the success of graft healing [9]. Rigid implants may also prevent gap closure following graft subsidence or contact osteolysis [5]. Thus, the necessity for additional anterior implants in unstable spinal fixation cases should be given serious consideration.

Bone grafts should be maintained under maximum compression to optimize fusion [6]. Many authors have indicated the importance of implant-mediated compression on strut grafts for healing [10,11]. It is therefore important to focus on the graft-bone interface in order to differentiate which fixation technique could best achieve the desired level of compression. Biomechanical studies also show that spinal ligament structures play an important role in restricting segmental movement and providing stability to implant constructs [12-14]. This is particularly important in severely unstable rotation/translation type C fractures [15], where there is often disruption of the longitudinal spinal ligaments. Approximately 20% of all spinal fractures demonstrate longitudinal ligament disruption, which is occasionally exceeded depending on case series in specialized centers [16,17].

To determine which technique would preserve the maximum compression and contact in the graft-vertebral bone interface, the aim of this *ex vivo* study was to investigate the compression and fixation capabilities of posterior, anterior and combined instrumentation with internal fixator and anterior implants on monosegmental strut graft repair, and to examine the role of severed spinal ligaments in these treatment strategies.

## Methods

**Figure 1:**
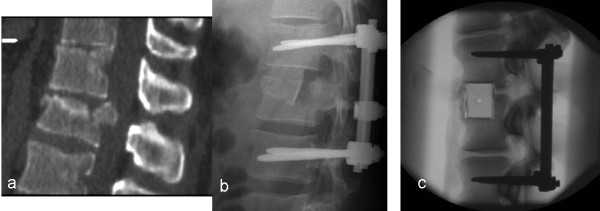
**Clinical background of the study.** Radiographs of a patient with an L1 fracture type A3.1 stabilized with an internal fixator and “pressfit” anterior strut autograft from the iliac crest. **a)** preoperative CT; **b)** postoperative fixation; **c)** analogous study specimen (calf spine). For experiments, thoracolumbar calf spine segments were stabilised posteriorly by internal fixator. The equivalent of a cranial burst zone was then cut with the help of a template to simulate an idealized notch within which the block graft was impacted.

Fresh-frozen thoracolumbar calf spines (E. Schmidt & Son, inc., Neunkirchen, Germany) were immediately used after thawing and preparation, in which surrounding soft tissue and muscles were dissected with care to preserve bone, discs and spinal ligaments. An incomplete burst fracture model, representing the Magerl/AO type A3.1 fracture [15] (Figure 1a), was created to simulate the fracture zone. This was done by resecting the cranial part of L1, including its posterior wall section and cranial disc, but leaving the anterior longitudinal ligament intact. The resection borders were kept strictly parallel through the use of a template and an oscillating saw to reflect an ideal intraoperative situation before insertion of the block graft.

**Figure 2:**
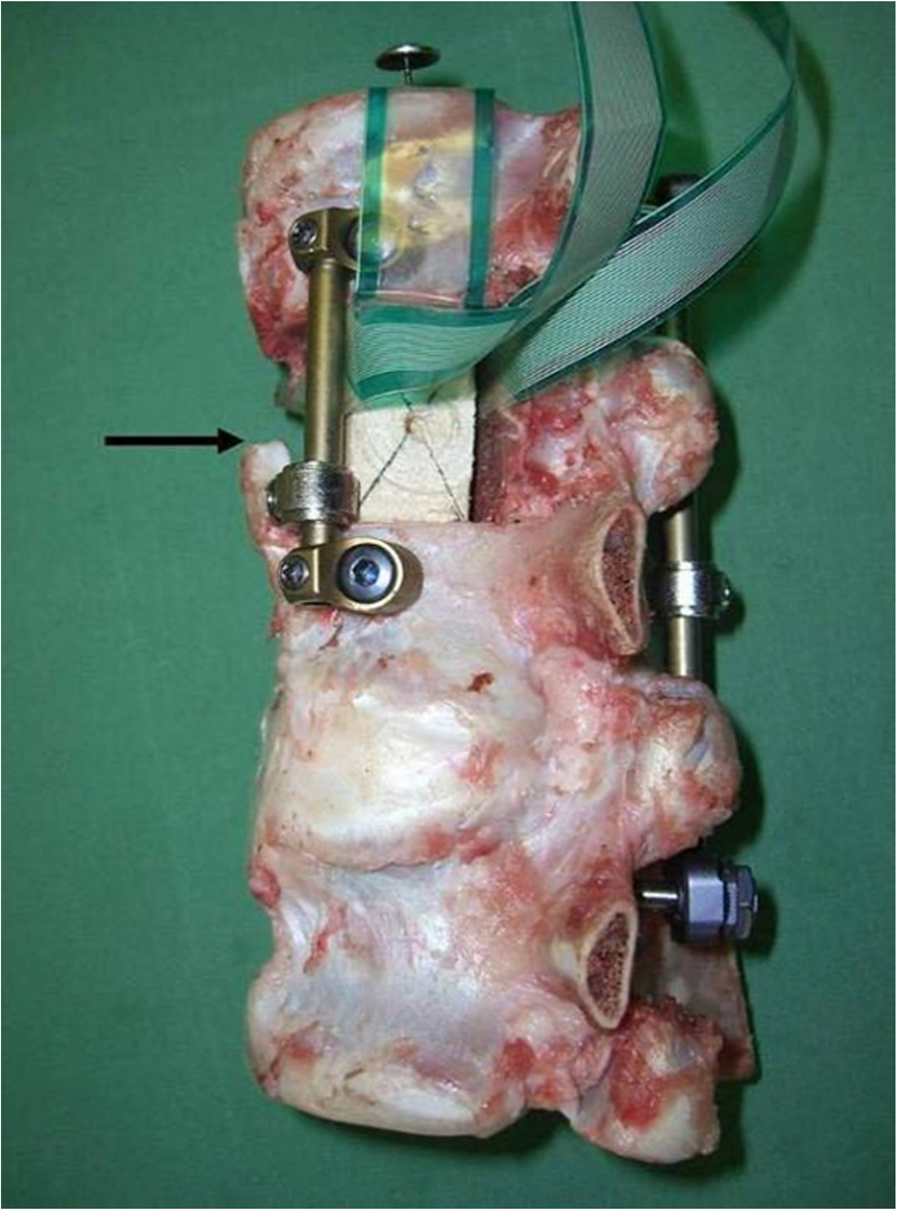
**Stabilised calf spine specimen, with block as a strut graft substitute and sensor *in situ*, showing the combined fixation method by posterior fixator (“USS” Internal Fixator**^**™**^**, Synthes®) and anterior rod (Ventrofix**^**™**^**, Synthes®) under compression.** This setup illustrates an experiment of group C-Type, in which all ligamentous connections were transected to represent an AO type C injury. (Arrow: separated anterior longitudinal ligament).

To investigate the influence of the injury pattern on bone graft measures, we randomly assigned specimens into two groups of different fracture types, according to the Magerl/AO spinal fracture classification [15]: group A-Type (n =8), as pure compression injuries treated as described above, and group C-Type (n = 8), which represented fractures combined with ligamentous injury that caused rotational/translation instability. In the group C-Type, all of the soft tissues (anterior and posterior longitudinal ligaments, ligamentum flavum and interspinal ligaments; facet joint capsules) were transected with a scalpel at the Th12/L1 level (Figure 2).

All specimens were stabilised according to standard protocols, with a posterior stabilizing internal fixator through transpedicular Schanz-screws (USS™-fracture Fixator®, Synthes, Oberdorf, Switzerland) or an anterior locking screw single rod construct (Ventrofix®, Synthes, Oberdorf, Switzerland) (Figure 2). Normal lordosis and interbody spacing were maintained in the specimens to simulate a hypothetical reduction.

Iliac crest autografts were simulated by wooden blocks (20 × 30 mm) cut to an appropriate gap length, with a 1 mm overhang to create a “pressfit” situation that reflects the method used in the clinical setting (Figure 1b,c). The specimens were kept moist with saline solution spray during the course of the experiments at constant room temperature.

Compression force and the contact area on the grafts were measured with thin, electro-resistive sensor films (sensor model 5033, Iscan® Tekscan Inc., South Boston, MA) that had been inserted into the defect site before the block grafts were impacted. New sensors were used for each series of experiments to minimize the effect of sensor deterioration. Conditioning and calibration was performed according to the manufacturer’s recommendations and methodology, as described previously [16,18]. The accuracy and reproducibility of this system have been reported in prior studies [19,20].

Compressive force (N) and contact area (mm^2^) of the grafts were recorded online for 3 sec. with sensor scanning at 100 Hz. The signals were averaged and processed by the Iscan® software during the setup conditions that reflect surgical practice, as follows:

IF Pressfit graft impaction into the defect on internal fixator stabilized specimens.

AR/IF As previous, but with supplemental anterior compression by Ventrofix® single rod implant.

IF + PC After posterior compression by internal fixator only, with no anterior implant.

AR Anterior compression by anterior single rod stabilisation alone (Ventrofix®).

This allowed investigation of the four common surgical applications for anterior strut graft fixation: by (1) an internal fixator, (2) anterior interbody implant-mediated compression in addition to an internal fixator, (3) posterior interbody compression by the internal fixator or (4) anterior interbody compression by the anterior compressing implant only. Instrumentation and compression techniques were performed following the manufacturers’ instructions and manuals by an experienced surgeon.

The collected data for compressive force and contact area were calculated as the mean and standard deviation (in parentheses). After testing for normality, these values were then compared for statistical significance by t-test and using repeated measures analysis of variance with multiple comparisons tests (Holm-Sidak). Significance was set at *P* < 0.05, as determined by statistical software analysis (SigmaStat 3.5, Systat®, San Jose, CA).

## Results

**Figure 3:**
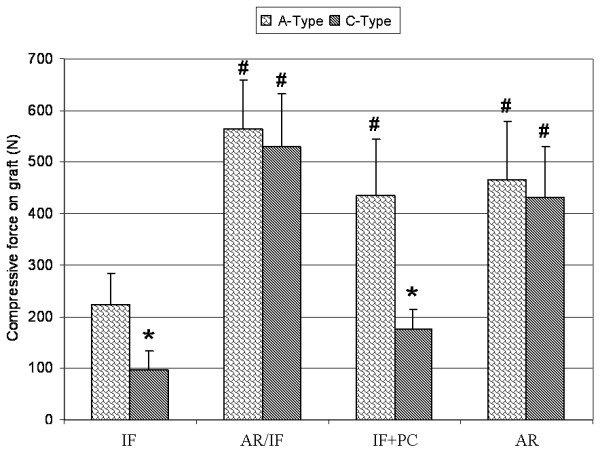
**Compressive forces on the block graft under various stabilization techniques for type A and type C injuries.** Results are presented as the mean (SD). Significant differences: # vs. IF: *P* < 0.05; * vs. the corresponding fixation in group A-Type *P* < 0.001. Note: anterior fixation alone for type C injuries was for experimental purpose only and should not be performed clinically.

In calf spines with intact soft tissue (group A-Type impaction), the 1 mm-oversized wooden dowels provided a compression of 224 (59) N with posterior stabilization by an internal fixator (IF). The resulting contact area in the interface between the graft and the resection edge of the vertebral body reached 449 (72) mm^2^, representing ¾ of the maximal calculated surface of a block graft (20 × 30 mm). Block graft compression could be increased by more than 2-fold of this “pressfit” baseline by the use of additional fixation on the anterior column with the rod system and anterior compression (AR/IF) or with compression using the posterior fixator (IF + PC) (Figure 3). This significantly improved the graft contact area, reaching roughly 90% of the maximum surface (Table 1). Compression to the graft by the anterior rod implant alone (AR) generated compressive forces around the same magnitude (490 (68) mm^2^), but the graft contact area was only 82% of the maximum surface area; however, this was not significantly different to IF stabilisation alone.

**Table 1:** Contact area on the grafts in the 2 groups A-Type and C-Type showing the effects of different fixation techniques from posterior, anterior or combined instrumentations

***Group***	**IF**	**AR/IF**	**IF + PC**	**AR**
**A-Type**				
**Mean (mm**^**2**^**)**	449	540 ^#^	533 ^#^	490
**SD**	72	67	45	68
***% of max. (SD)***	*75 (12)*	*90 (11)*	*89 (8)*	*82 (11)*
**C-Type**				
**Mean (mm**^**2**^**)**	318 *	521 ^#^	308 *	435
**SD**	90	60	88	89
***% of max. (SD)***	*53 (15)*	*87 (14)*	*51 (15)*	*73 (15)*

Mean and Standard deviation, percentage calculated on the base of a theoretical maximum of 600 mm^2^ on the graft. # vs. IF in the same group *P* < 0.05, * vs. corresponding fixation in group A-Type p < 0.01.

**Figure 4:**
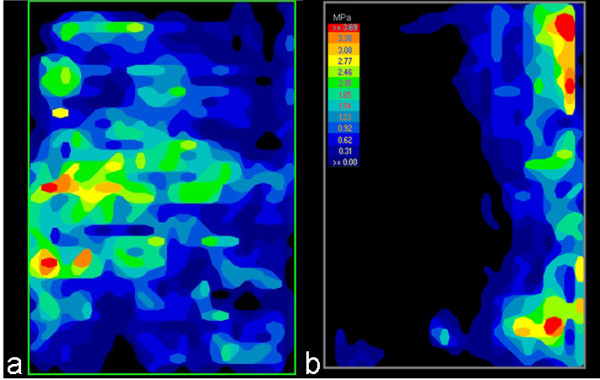
**Examples of pressure film recordings with block grafts impacted in the anterior intercorporal notch with posterior instrumentation (IF) in (a) an example of A-Type and (b) an example of C-Type group tests.** Orientation of the image: left = anterior; right = posterior. Note the absence of contact on the anterior part of the graft due to the lack of tension banding in the C-type injury.

When the surrounding ligaments and capsule-tissue were severed (group C-Type), the “pressfit” method of insertion by impacting the blocks was unable to reach the compressive forces obtained in the group A-Type. At baseline (IF), the group C-Type injury model had a compression of 97 (36) N and a resulting contact area of 318 (90) mm^2^; both measurements were significantly lower than the initial values obtained in the group A-Type (Figure 3). Indeed, the graft contact area under these circumstances was only 50% of the maximum obtainable contact area (Table 1, Figure 4a,b). These effects could be effectively countered with AR/IF, which induced a compressive force that was similar to that in group A-Type (Figure 3). This measure was also coupled with an increased contact area of 521 (60) mm^2^ (87%) (Table 1).

In contrast, the sole posterior compression technique (IF + PC) failed to substantially increase the compressive force or the contact area on the interbody block graft in the group C-Type injury model (Figure 3). Exclusive anterior instrumentation and compression (AR) in group C-Type, without classic posterior fixation, however, increased the compressive force as compared to the impacted technique, but the increase in the graft contact area did not reach statistical significance.

## Discussion

This study was performed on calf spines, which are regarded valid substitutes for human cadaveric spine testing experiments [21]. Human spines are not only more difficult to obtain and expensive, but have a high variability in bone mineral density and degeneration, resulting in data scattering, which may interfere with a correct interpretation of the results. Our fracture model, similar to that published previously [13,22-24], preserves the ligaments that influence spinal stability and, to our knowledge, has provided for the first time the use of electro-resistive pressure-sensitive films to investigate continuously the interface between the strut grafts and the vertebral body. In simulated compression type A fractures with posterior stabilization (using an internal fixator), an impaction of 1 mm-oversized block grafts provided graft compression and a contact area that represented 75% of the maximum possible surface on the wooden dowels. While this compression represents a respectable force for such a simple technique that is commonly used for anterior fusion, the reduced contact area reflects the real-life difficulties of fitting bone grafts: cutting parallel lines that correspond with resection planes. Even under the given optimal wet lab circumstances, study protocols, and available templates, it was rarely possible to obtain full contact on the block grafts. This reduced contact area could be improved to near maximal values by either compression with an additional anterior implant or by shortening the posterior fixator itself (few millimeters) for the common type A compression injuries. Interestingly, this could not be fully replicated with the use of an anterior rod construct alone (AR). Each one of these techniques is practiced in surgery to improve the grafting result and provide adequate fusion [3,25,26]. However, severed surrounding soft tissue, which corresponds to the rotation/translational injuries of type C fractures, can have a profound effect on the “pressfit” impacted technique, as shown in our experiments: the block graft compression and contact area were reduced by 50% as compared with those measurements in the group A-Type fractures. Furthermore, posterior compression of the internal fixator (IF + PC) did not improve these results, likely caused by the lack of the tension banding of the anterior ligament and the absence of supporting anterior implants. The posteriorly prevailing pattern of contact and the pressure distribution backwards was reflecting the excentric effect of the posterior Internal Fixator device. In order to increase the compression and graft contact in the group C-Type, an anterior implant, acting directly on the anterior column, was mandatory. An exclusively anterior fixation (AR) of the group C-Type injury model did not induce positive effects, and it is important to emphasize that anterior fixation of type C fractures, without posterior stabilization was only used for experimental purpose; it is not recommended for clinical use.

Our findings underline the importance of ligaments and stabilizing soft tissue around the spine [27,28]. The differences are likely to be associated with the tension banding effects of the longitudinal ligaments, especially the anterior ligament, which had been transected to create the type C injury. Several authors have found these longitudinal ligaments contribute to the stability of the fixation and the fusion results [12,14,29]. Our results are consistent with the previous publication of the basic compression methods using an internal fixator [30], which showed that eccentric posterior compression does not simply transfer to the bone grafts placed in the anterior column. In addition, our studies represent a valuable extension to these findings by introducing the possibility of anterior implant-mediated compression and the importance of the ligaments in different types of injury.

When considering the dynamic role of the ligaments in the stabilised spine, our results allow supplemental aspects to the following studies, which stress the importance of graft compression forces under movement and compression versus contact area in different setups. Extension movements of instrumented spines have been shown to reduce the compressive force on interbody grafts in cadaveric human corpectomy specimens in the spine simulator with pure bending moments [7]. From the biomechanical point of view, this would endorse the surgical methods of adding anterior implants as a combined (anterior/posterior) fixation technique to improve graft healing. When considering our results, it remains unclear what influence the preservation of ligaments or the application of pretension to the implants has in this experimental setup. Since maintaining the graft compression is desired in all types of movement, including extension, it may be concluded that an adequate pretension to an oversized graft and an intact anterior longitudinal ligament could have beneficial effects on healing, even without additional implants placed anteriorly.

The effect of internal fixator pretension was investigated by a finite element analysis of bone graft techniques in a computer simulation [31], in which physiological loading of the spine and follower loads were considered. The relative contact areas of the grafts of 60-80% in the “standing” position dropped to 10-20% of the contact under extension movement. Pretension of the posterior implant (internal fixator), by reducing the screw distance, as performed clinically during surgery for “posterior compression”, paradoxically caused a decrease in the compressive force on the graft, analogous to our results (IF + PC of group C-Type). Indeed, in this finite-element model, the anterior ligaments and anterior annulus parts of the disc were omitted. We observed similar results with group C-Type in our study. Sparing anterior tension banding, similar to the situation of the group A-Type in our study, could possibly add to the graft contact area under different movements.

The maintenance of implant-mediated graft compression is of major importance to ensure healing. Previous clinical experience with cervical anterior autograft fusions has endorsed this philosophy and encouraged the further investigations of aspects on bone-graft interactions [32]. Bone autograft incorporation and fusion in the spine are undoubtedly dependent on biological factors as well as the influence of drugs (non-steroidal anti-inflammatory), nicotine [33-35] or perfusion quality [36]. Other investigations have focused on the size and contact area of the bone grafts, which seems to be of equal importance for a successful fusion [37,38] and the avoidance of resorption. In these cases, the role of stiffness is still uncertain, since transmission of stress to the grafted bone is crucial for fusion and remodeling. Rigid fixation, as achieved with combined anterior/ posterior instrumentation, could eliminate the transmission of load needed for bone fusion and may actually reduce bone healing [27,39-41]. It has not been established how much residual segmental mobility or micromotion at the interface between the host bone and the implant/ bone graft can be tolerated [42].

In this regard, the results of our study could influence the choice of strut graft fixation for patients. “Pressfit” impaction or simple posterior compression by the internal fixator provides good contact and compression to the anterior strut grafts in fractures without ligament deficiency. This would subsequently avoid the requirement for supplemental implants and excessively rigid fixation (so-called 360° fixation). On the other hand, for cases that present with ligamentous injuries, as in Magerl /AO type C fractures, it seems necessary to add anterior compressing implants to secure the bone grafts. The limitation of this bench-top study is that the findings from an unloaded bovine ex-vivo model might not necessarily be fully applicable to a scenario in-vivo.

## Conclusions

Our experimental study used the modern generation of thin, pressure-sensitive films to investigate for the first time the interface between interbody graft and vertebral bone in a stabilised fracture model. This new approach for the assessing the bone graft/vertebral body interface could help to clarify the requirements for fixation. Our results may provide knowledge that can help surgeons in their choice of implant and surgical approach for successful spinal reconstruction.

## Abbreviations

AO: Arbeitsgemeinschaft für osteosynthesefragen; USS™: Universal spine system, brand name for the internal fixator implant; IF: Impacted block graft, spine fixation by an Internal Fixator, posteriorly; AR/IF: Additional graft compression by anterior rod implant, fixation by an Internal Fixator; IF + PC: Graft compression through Internal Fixator posteriorly, no anterior implant; AR: Anterior graft compression and fixation by anterior rod implant, without an Internal Fixator.

## Competing interests

The authors declare that they have no financial or non-financial competing interests.

## Authors’ contributions

AP conceived the study, participated in its design and drafted the manuscript. JHH participated in the design of the study and helped to draft the manuscript. FV carried out the experiments and performed the statistical analysis. MB revised the manuscript critically for important intellectual content. TP made substantial contributions to the conception and design of the study and helped in data analysis and interpretation. All authors read and approved the final manuscript.

## Pre-publication history

The pre-publication history for this paper can be accessed here:

http://www.biomedcentral.com/1471-2474/14/254/prepub
